# Supplementary material for: Ungulates’ Behavioral Responses to Humans as an Apex Predator in a Hunting-Prohibited Area of China
Source: Animals (Basel). 2023 Feb 25;13(5):845. doi: 10.3390/ani13050845 (PMC10000205; doi:10.3390/ani13050845)
Supplement: Supplementary file 1 [file animals-13-00845-s001.zip › animals-2203150-supplementary.pdf]

Supplementary Materials

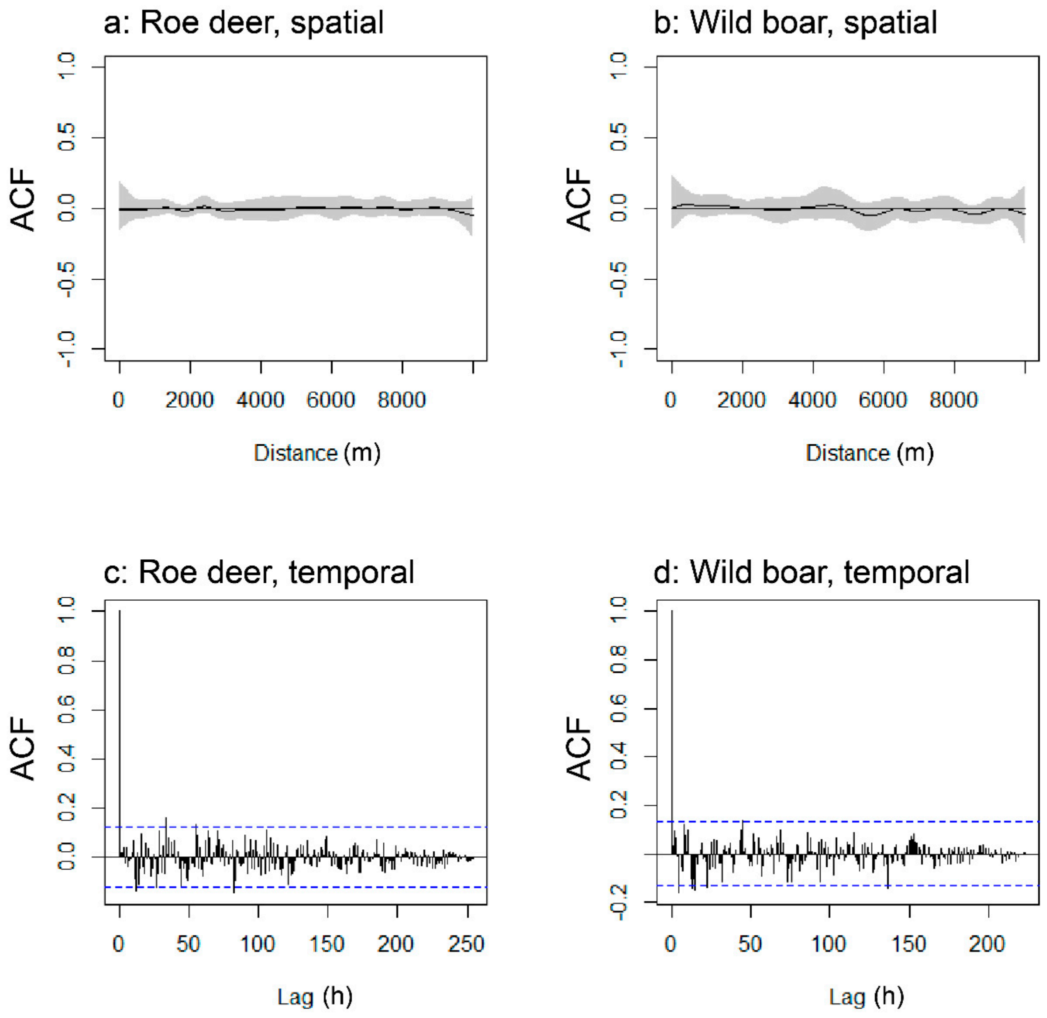

Figure S1. Spatial and temporal auto-correlation functions (ACF) of the residuals of the best logistic regression models for the flight probability.

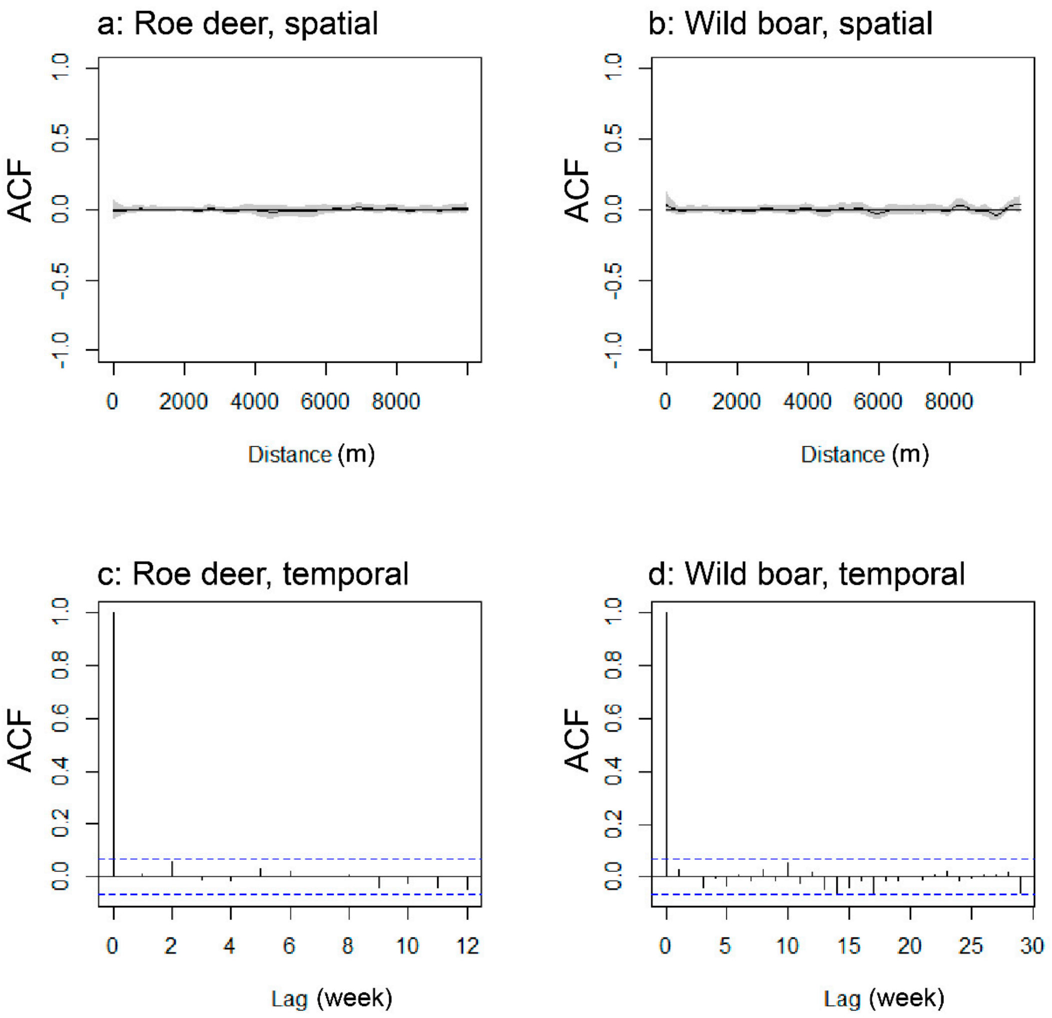

Figure S2. Spatial and temporal auto-correlation functions (ACF) of the residuals of the best logistic regression models for the detection probability.
